# SHFL Post-Transcriptionally Restricts Coxsackievirus A16 In Vitro and In Vivo

**DOI:** 10.3390/v18020192

**Published:** 2026-01-31

**Authors:** Huijie Li, Rui Wang, Jichen Li, Wei Duan, Yucai Liang, Qiang Sun, Jianfang Zhou, Yong Zhang

**Affiliations:** 1 National Key Laboratory of Intelligent Tracking and Forecasting for Infectious Diseases (NITFID), National Institute for Viral Disease Control and Prevention, Chinese Center for Disease Control and Prevention, Beijing 102206, China; lihuijie_jiejie@163.com (H.L.); ruiwang_97@163.com (R.W.); jichenli666@163.com (J.L.); duanwei0617@126.com (W.D.); yucai.liang@hotmail.com (Y.L.); 2 National Polio Laboratory, National Institute for Viral Disease Control and Prevention, Chinese Center for Disease Control and Prevention, 155 Changbai Road, Beijing 102206, China; 3 National Health Commission Key Laboratory of Microbial Genomics, National Health Commission Key Laboratory of Laboratory Biosafety, National Institute for Viral Disease Control and Prevention, Chinese Center for Disease Control and Prevention, 155 Changbai Road, Beijing 102206, China; 4 World Health Organization Polio Reference Laboratory for the Western Pacific Region, National Institute for Viral Disease Control and Prevention, Chinese Center for Disease Control and Prevention, Beijing 102206, China

**Keywords:** coxsackievirus A16, post-transcriptional restriction, innate antiviral immunity, viral RNA stability, enterovirus replication, neurotropism

## Abstract

Coxsackievirus A16 (CVA16), a major etiological agent of hand, foot, and mouth disease, is increasingly contributing to neurological complications, with no vaccines or virus-specific antivirals currently available. To identify CVA16-restricting host factors, we investigated the role of the interferon-stimulated gene shiftless (*SHFL*), previously implicated in the control of other RNA viruses. Using CRISPR–Cas 9, we generated *SHFL* knockout rhabdomyosarcoma cells and assessed viral replication, cytopathic effects, and replication stage dynamics. We evaluated disease progression and tissue injury in neonatal mice infected with a mouse-adapted CVA16 strain. *SHFL* expression was strongly induced during CVA16 infection and was inducible by exogenous interferon-β treatment, and its loss markedly increased infectious virus production, accelerated early replication, and exerted severe cytopathic effects. In vivo, *SHFL* deficiency led to rapid weight loss, pronounced neurological signs, increased viral burden across multiple tissues, and uniform mortality, together with high viral loads and extensive pathological damage in the central nervous system, lungs, and skeletal muscle. Transcriptomic analyses revealed *SHFL*-dependent modulation of adhesion- and mitogen-activated protein kinase-related pathways. Overall, our results suggest *SHFL* as a key determinant of host resistance to CVA16, acting mainly at the post-transcriptional stage to limit viral spread and tissue injury, and highlight *SHFL*-linked pathways as promising host-directed antiviral targets.

## 1. Introduction

Recently, coxsackievirus A16 (CVA16), a principal etiological agent of hand, foot, and mouth disease, has been showing increasing prevalence across the Asia–Pacific region, with reports indicating partial displacement of enterovirus (EV)-A71 during outbreaks [1,2,3]. A regional surveillance study in Shenyang from 2013 to 2023 provided further insight into CVA16 evolution and persistence in northeastern China [4]. Although CVA16 infection is often self-limiting, it exhibits neurotropic potential and can lead to paralysis, brainstem dysfunction, and severe neurological inflammation driven by pathways such as the Toll-like receptor 2–MyD88–tumor necrosis factor-α pathway [5]. Notably, no licensed cross-protective vaccines or specific antiviral agents targeting CVA16 are currently available [6]. Supporting the neurotropic nature of enteroviruses, murine oral CVA16 models have demonstrated systemic and neuromuscular pathologies, underscoring the relevance of in vivo modeling [7]. Despite substantial nucleotide variations among its circulating strains, several coding regions remain highly conserved at the amino acid level, suggesting evolutionary constraints that preserve essential viral functions while enabling immune evasion [8,9,10]. Therefore, identifying host determinants limiting CVA16 replication is critical to develop novel host-directed therapeutic strategies [11].

Interferon (IFN)-stimulated genes (ISGs) constitute a central component of the innate antiviral defense system [12]. Specifically, shiftless (*SHFL*) acts as a broad-acting antiviral restriction protein [13,14]. Regulated by the Janus kinase–signal transducer and activator of transcription pathway and encoded on chromosome 19p13.2, *SHFL* inhibits several RNA viruses, including human immunodeficiency virus-1, flaviviruses, and EV-A71 [15,16]. Its reported action mechanisms include suppression of −1 ribosomal frameshifting, modulation of viral RNA stability, and degradation of viral polymerase proteins [17,18,19]. For EV-A71, *SHFL* targets the 3D polymerase via conserved domains, including a zinc-finger motif and residues 164–199, leading to ubiquitin–proteasome-mediated degradation of the replication complex [20,21]. These observations suggest that *SHFL* also restricts CVA16, which shares highly conserved RNA-dependent RNA polymerase domains with EV-A71 [22].

Type I IFN signaling, particularly via the Toll-like receptor 3–TIR-domain-containing adapter-inducing IFN-β axis, is implicated in the control of CVA16 infection [23]. Considering the structural conservation of enteroviral polymerase proteins and established antiviral role of *SHFL* against EV-A71, we hypothesized that *SHFL* restricts CVA16 through a similar mechanism, possibly by acting at the post-transcriptional stage of the viral life cycle [21,22]. Furthermore, cytokine responses triggered during CVA16 infection, including tumor necrosis factor-α-mediated innate signaling, may contribute to *SHFL* induction through interferon-dependent or interferon-independent mechanisms [5]. In this study, we explored the functional stage at which *SHFL* restricts CVA16 replication and characterized its impact on viral pathogenesis. Our findings provide key insights into host–EV interactions and can facilitate the development of new host-directed antiviral approaches for hand, foot, and mouth disease.

## 2. Materials and Methods

### 2.1. Ethics Statement

All animal experiments adhered to the guidelines and were approved by the Ethics Review Committee of the National Institute for Viral Disease Control and Prevention, Chinese Center for Disease Control and Prevention (approval no. 20201022059; approval date: 29 October 2020). Wild-type (WT) C57BL/6 mice were purchased from SPF Biotechnology Co., Ltd. (Beijing, China), and *SHFL* knockout (KO) mice were obtained from Cyagen Biosciences (Guangzhou, China). All animals were housed at the Animal Center of the Chinese Center for Disease Control and Prevention under specific pathogen-free (SPF) conditions in individually ventilated cages, with ad libitum access to food and water. All procedures were performed in a pathogen-free environment to ensure animal welfare and experimental reliability.

### 2.2. Cells and Virus

Human rhabdomyosarcoma (RD) cells (ATCC, Manassas, VA, USA) were cultured in minimal essential medium supplemented with 10% fetal bovine serum (Gibco, Grand Island, NY, USA) at 37 °C in a 5% CO_2_ atmosphere. The Coxsackievirus A16 (CVA16) strain HB2010-114, originally isolated from Hebei Province (Cangzhou City, China), was propagated and titrated in RD cells.

To generate a mouse-adapted CVA16 strain, 2-day-old ICR mice were intramuscularly inoculated with wild-type (WT) virus and monitored daily for clinical signs. Brain tissues collected at 4 d post-infection (dpi) were homogenized in phosphate-buffered saline containing 1% penicillin–streptomycin (HyClone, Logan, UT, USA) and centrifuged at 10,000× *g* for 10 min at 4 °C. The clarified supernatant was used to infect the RD cell monolayers. Upon induction of pronounced cytopathic effects, the infected cells were subjected to three freeze–thaw cycles, followed by centrifugation at 10,000× *g* for 10 min at 4 °C, and filtered through a 0.22 μm membrane. The resulting filtrate was intramuscularly administered to 3-day-old ICR mice. After five consecutive passages, a stable mouse-adapted strain (designated CVA16-P5) was obtained, which consistently induced infection in 3-day-old C57BL/6 mice.

### 2.3. RNA Extraction and Reverse Transcription-Quantitative Polymerase Chain Reaction (RT-qPCR)

Total RNA was extracted from CVA16-infected RD cells using the Tianlong Nucleic Acid Extraction Kit (Tianlong Science and Technology Co., Ltd., Xi’an, China). cDNA synthesis and amplification were performed with the UniPeak U+ One Step RT-qPCR SYBR Green Kit (Vazyme Biotech Co., Ltd., Nanjing, China) using 1 µg of input RNA. qPCR was performed on the QuantStudio 5 Real-Time PCR System (Thermo Fisher Scientific, Waltham, MA, USA) to quantify ISGs, including *SHFL*, and viral *VP1*. Glyceraldehyde-3-phosphate dehydrogenase served as an endogenous control. Relative expression was calculated using the 2^−ΔΔCt^ method. *VP1* RNA levels were measured using a probe-based assay. All reactions were performed in triplicate. All primer and probe sequences are listed in Table S1.

### 2.4. Generation of SHFL Knockout (KO) RD Cells Using the Clustered Regularly Interspaced Palindromic Repeat (CRISPR)–CRISPR-Associated Protein 9 (Cas9) System

*SHFL* KO RD cells were generated using the CRISPR–Cas9 system. Single guide RNA pairs targeting the *SHFL* coding exon (sense, 5′-CACCGTGCAGCCTCACCGTACACGA-3′; antisense, 3′-CACGTCGGAGTGGCATGTGCTCAAA-5′) were cloned into the BsmBI site of the lentiCRISPR v2 vector (Addgene plasmid #52961; Addgene, Watertown, MA, USA). Lentiviral particles were produced by transfecting HEK293T cells, after which RD cells were transduced in the presence of 8 µg/mL polybrene (Sigma-Aldrich, St. Louis, MO, USA). The cells were selected with 2 µg/mL puromycin (InvivoGen, San Diego, CA, USA) for seven days, and single-cell clones were isolated via flow cytometry. Clones were screened by Western blot analysis, and a monoclonal line with no detectable *SHFL* protein under the experimental conditions used was selected for subsequent experiments. Clone #19 harbors CRISPR/Cas9-induced frameshift mutations in the *SHFL* coding region, resulting in disruption of the open reading frame and functional inactivation of *SHFL*.

### 2.5. Western Blotting

Total protein was extracted using the radioimmunoprecipitation assay buffer (RIPA Lysis Buffer, Cat# P0013B; Beyotime Biotechnology, Shanghai, China) with protease inhibitors. Protein concentration was determined via bicinchoninic acid assay (Cat# P0010; Beyotime Biotechnology, Shanghai, China). Equal amounts of proteins were resolved via sodium dodecyl sulfate-polyacrylamide gel electrophoresis and transferred to polyvinylidene difluoride membranes (Immobilon-P PVDF Membrane, 0.45 µm, Cat# IPVH00010; Millipore, Burlington, MA, USA). After blocking with 5% non-fat milk (Skim Milk Powder, Cat# 232100; BD Difco™, Franklin Lakes, NJ, USA), the membranes were incubated overnight with an anti-*SHFL* antibody (1:1000; Cat# HPA042001; Sigma-Aldrich, St. Louis, MO, USA) at 4 °C. After incubation with a conjugated secondary antibody (1:5000; Cat# ab6721; Abcam, Cambridge, UK), signals were visualized using an enhanced chemiluminescence system (Cat# 1708280; Bio-Rad Laboratories, Hercules, CA, USA).

### 2.6. Cell Viability Assay

Cell viability was assessed via cell counting kit-8 assay (CCK-8; Cat# CA1210; Solarbio Life Sciences, Beijing, China). Human rhabdomyosarcoma (RD) cells were seeded in a 96-well plate and incubated at 37 °C in a humidified 5% CO_2_ atmosphere for 24 h. After adding the cell counting kit-8 reagent (10 µL/well), the cells were incubated at 37 °C for 1–4 h. Absorbance at 450 nm was measured using a microplate reader (Multiskan™ FC Microplate Photometer; Thermo Fisher Scientific, Waltham, MA, USA).

### 2.7. Plaque Assay

RD and *SHFL* KO cells were seeded in 6-well plates until they reached confluence. Serial 10-fold dilutions of CVA16 (10^−2^ to 10^−6^) were added to the cells (50 µL/well), followed by incubation with gentle rocking at 37 °C for 1 h. After removing the inoculum, the cells were overlaid with the Dulbecco’s modified Eagle’s medium containing 1.2% Avicel and 2% fetal bovine serum. At 72 h, the cells were fixed with 4% paraformaldehyde and stained with 0.5% crystal violet. After drying at room temperature, the number of plaques was counted manually with the naked eye.

### 2.8. One-Step Growth Curve

WT and *SHFL* KO cells were infected with CVA16 at a multiplicity of infection of 5 for 1 h at 37 °C. After phosphate-buffered saline (PBS; Gibco, Grand Island, NY, USA) washing, fresh medium was added, and the samples were collected at 2, 4, 6, 8, 10, 12, 24, and 48 h post-infection (hpi). The supernatants were used for 50% tissue culture infectious dose (TCID_50_) titration (Reed–Muench method). Intracellular RNA was extracted using TRIzol (Cat# 15596026; Invitrogen, Carlsbad, CA, USA), reverse-transcribed, and quantified via VP1-specific probe-based RT-qPCR.

### 2.9. Immunofluorescence Assay

RD cells were seeded onto sterile glass coverslips placed in 24-well plates and infected with CVA16 at the indicated multiplicity of infection (MOI). At 24 h post-infection, cells were washed twice with phosphate-buffered saline (PBS) and fixed with 4% paraformaldehyde for 15 min at room temperature. After fixation, cells were permeabilized with 0.1% Triton X-100 in PBS for 10 min and subsequently blocked with 5% bovine serum albumin (BSA) in PBS for 1 h at room temperature.

Cells were then incubated overnight at 4 °C with a mouse polyclonal anti-CVA16 antiserum, which was generated by immunizing mice with purified CVA16 virions and was the same antibody used for immunohistochemical analysis, diluted 1:100 in blocking buffer. After washing with PBS, cells were incubated with Alexa Fluor 488–conjugated goat anti-mouse IgG secondary antibody (1:100; Invitrogen, Carlsbad, CA, USA) for 1 h at room temperature in the dark. Nuclei were counterstained with DAPI. Coverslips were mounted using antifade mounting medium, and fluorescence images were acquired using a fluorescence microscope.

### 2.10. Mouse

*SHFL* knockout (*SHFL*-KO) mice were obtained from Cyagen Biosciences (Guangzhou, China; Model No. S-KO-09133). The mice were generated on a C57BL/6 genetic background using CRISPR/Cas9-mediated genome editing. Wild-type (WT) C57BL/6 mice were used as controls in all in vivo experiments.

### 2.11. Mouse Infection

WT and *SHFL* KO C57BL/6 neonatal mice were intramuscularly inoculated with 50 µL of CVA16-P5 (10^6^ TCID_50_/50 µL). The mice were monitored daily for weight, survival, and clinical symptoms. Their tissues were collected at 4–6 days post-infection (dpi) for viral titer quantification and histopathological analyses.

### 2.12. Viral Titer Assay

Mouse tissues (heart, liver, spleen, lungs, kidneys, brain, skeletal muscle, and spinal cord) were collected at 1, 3, and 5 dpi. The samples were homogenized in the Dulbecco’s modified Eagle’s medium, centrifuged, serially diluted, and inoculated onto the RD cell monolayers. Cytopathic effects were monitored for 5–7 d, and viral titers were calculated using the Reed–Muench method and expressed as log_10_ TCID_50_/50 µL.

### 2.13. Histopathology

The tissues were fixed in 4% formalin for 24 h, embedded in paraffin, and sectioned at 5 µm. The sections were stained with hematoxylin and eosin (H&E). Brain tissues were subjected to Nissl staining to assess neuronal integrity. For immunohistochemistry, the sections were deparaffinized, rehydrated, and subjected to antigen retrieval, followed by blocking with 3% bovine serum albumin (BSA; Cat# ZLI-9056; ZSGB-Bio, Beijing, China). Slides were incubated overnight with anti-CVA16 polyclonal antiserum (prepared in-house by immunizing mice with purified CVA16-P0 viral particles) at 4 °C, followed by incubation with horseradish peroxidase-conjugated secondary antibody (1:1000; Cat# ab6789; Abcam, Cambridge, UK). Images were captured using the AxioCam MRc5 system (Carl Zeiss Microscopy GmbH, Jena, Germany).

### 2.14. RNA-Sequencing Library Preparation

Total RNA from RD and *SHFL* KO cells (n = 3/group) was extracted using TRIzol (Cat# 15596026; Invitrogen, Carlsbad, CA, USA). RNA concentration and integrity were measured using the Qubit 2.0 Fluorometer (Thermo Fisher Scientific, Waltham, MA, USA) and Bioanalyzer 2100 (Model G2939BA; Agilent Technologies, Santa Clara, CA, USA), with all samples showing RNA integrity number ≥ 8. Libraries were constructed via random priming and sequenced on the Illumina NovaSeq 6000 platform (Novogene Co., Ltd. Tianjin, China). Gene counts were generated using featureCounts (v1.5.0).

### 2.15. Bioinformatics Analysis

Quality control of raw reads was performed using FastQC (v0.11.9). Adapter trimming was performed using Trimmomatic (v0.39). The reads were aligned to the GRCh38 reference genome using STAR (v2.7.10a). Gene-level counts were obtained using featureCounts under the GENCODE v35 annotation. Differential expression was analyzed using DESeq2 (v1.30.1). Genes with adjusted *p* < 0.05 and |log_2_FC| > 1 were considered to be significantly differentially expressed. Gene Ontology and Kyoto Encyclopedia of Genes and Genomes pathway enrichment analyses were conducted using clusterProfiler (v4.0.5).

### 2.16. Statistical Analyses

Data were analyzed using GraphPad Prism 8.0 and ImageJ 1.51. Values are expressed as the mean ± standard deviation from at least three biological replicates. Survival curves were compared using the log-rank test. Tissue viral titers and Nissl quantification were analyzed via one-way analysis of variance, followed by Tukey’s post hoc test. Statistical significance was set at * *p* < 0.05, ** *p* < 0.01, and *** *p* < 0.001 (ns, not significant).

## 3. Results

### 3.1. CVA16 Infection Induces SHFL Expression, and Establishment of an SHFL KO RD Cell Model

**Figure 1:**
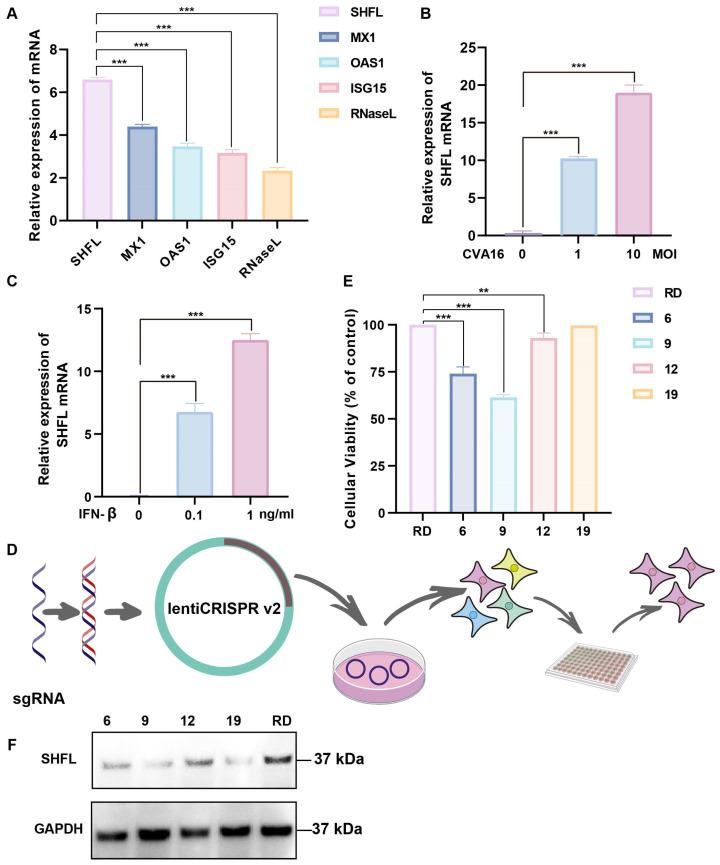
Induction of Shiftless (*SHFL*) by CVA16 infection and generation of *SHFL* knockout RD cells. (**A**) Relative mRNA expression levels of selected interferon-stimulated genes (ISGs) in RD cells following CVA16 infection, as determined by quantitative RT-PCR. (**B**) SHFL mRNA expression in RD cells infected with CVA16 at the indicated multiplicities of infection (MOIs), measured by quantitative RT-PCR. (**C**) *SHFL* mRNA expression in RD cells treated with exogenous IFN-β at the indicated concentrations. (**D**) Schematic illustration of the CRISPR–Cas9 strategy used to generate *SHFL* knockout (KO) RD cells. (**E**) Cell viability or growth characteristics of parental RD cells and monoclonal *SHFL* KO cell lines following single-cell sorting. (**F**) Western blot analysis of *SHFL* protein expression in parental RD cells and the selected *SHFL* KO clone (#19). Data are presented as mean ± standard deviation (SD; n = 3). ** *p* < 0.01, and *** *p* < 0.001.

*SHFL* was strongly induced in CVA16-infected RD cells, showing the highest mRNA upregulation among the ISGs examined, with levels exceeding those of MX dynamin-like GTPase 1, 2′-5′-oligoadenylate synthetase 1, *ISG15*, and RNase L (Figure 1A). *SHFL* expression increased in a dose-dependent manner with increasing multiplicities of infection (Figure 1B). Consistently, recombinant IFN-β treatment elevated *SHFL* expression in a concentration-dependent manner, indicating that IFN-β is sufficient to induce *SHFL* expression (Figure 1C).

To determine the functional contribution of *SHFL*, an *SHFL* KO RD cell line was generated using the CRISPR–Cas9 lentiviral system (Figure 1D). Several monoclonal cell lines were obtained following single-cell sorting, and clone #19 was selected based on its viability profile, which was closest to that of parental RD cells (Figure 1E). Western blot analysis revealed no detectable *SHFL* protein in clone #19 under the experimental conditions used, supporting its designation as a functionally *SHFL*-deficient and homogeneous cell model for subsequent experiments (Figure 1F).

### 3.2. SHFL Deficiency Promotes CVA16-Induced Cytopathology and Viral Replication

**Figure 2:**
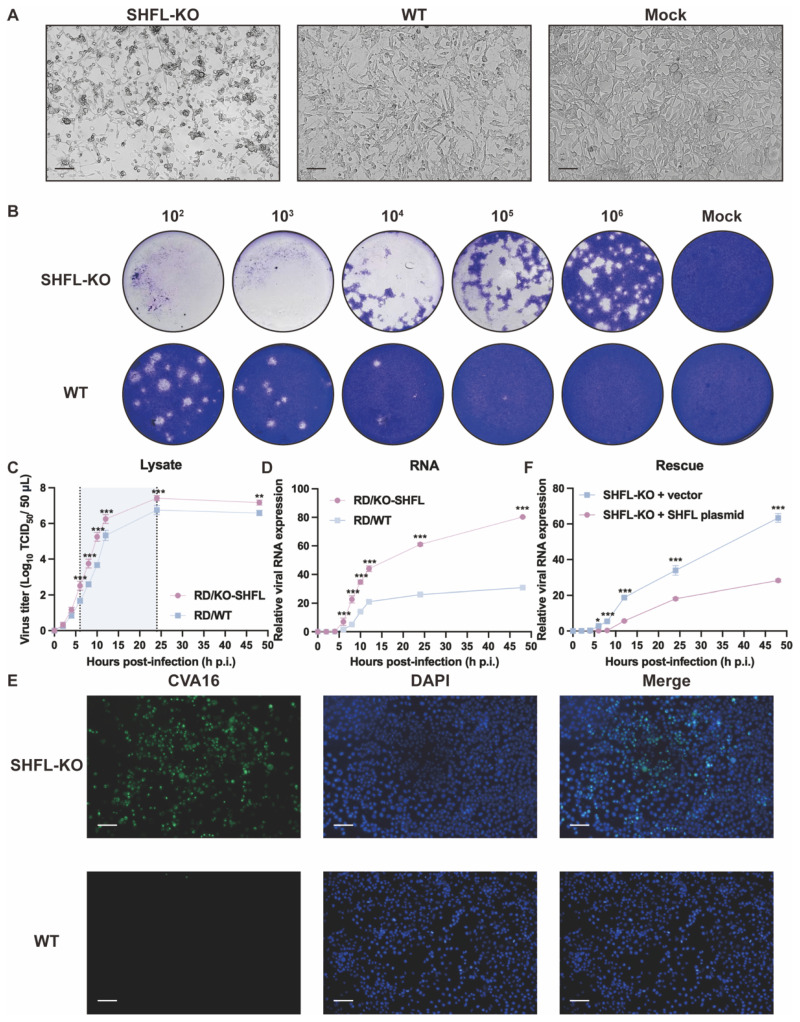
Analysis of CVA16 infection in *SHFL* knockout and wild-type RD cells. (**A**) Cytopathic effects observed in *SHFL* knockout (*SHFL* KO), wild-type (WT), and mock-infected RD cells at 24 h post-infection (hpi). Scale bars represent 200 μm. (**B**) Plaque assay analysis of CVA16 produced from *SHFL* KO and WT RD cells. Culture supernatants collected from infected cells were serially diluted and subjected to plaque formation assays. (**C**) One-step growth curve of CVA16 in *SHFL* KO and WT RD cells determined by TCID_50_ assay at the indicated time points post-infection. The shaded background highlights the time window with the greatest differences between groups. (**D**) One-step growth curve based on viral RNA quantification. Relative CVA16 viral RNA levels were measured by quantitative RT-PCR at the indicated time points post-infection. (**E**) Immunofluorescence staining of CVA16-infected *SHFL* KO and WT RD cells at 24 h post-infection. Scale bars represent 200 μm. (**F**) Rescue experiment in *SHFL* KO RD cells. Cells were transiently transfected with a *SHFL* expression plasmid or empty vector and subsequently infected with CVA16. Viral replication was assessed by quantitative RT-PCR analysis of viral RNA levels. Data are presented as mean ± standard deviation (SD; n = 3). * *p* < 0.05, ** *p* < 0.01 and *** *p* < 0.001.

To determine the functional consequences of *SHFL* loss during CVA16 infection, we compared infection outcomes between *SHFL* KO and WT RD cells. *SHFL* KO monolayers exhibited pronounced cytopathic changes as early as 24 hpi, characterized by extensive cell rounding, shrinkage, and detachment, whereas WT cultures retained a largely intact fibroblast-like morphology, similar to mock-infected cells. This striking contrast suggests that the absence of *SHFL* substantially sensitizes RD cells to CVA16-induced cytopathicity (Figure 2A).

Consistent with the observed morphological differences, plaque assays performed using supernatants from infected cultures revealed markedly elevated production of infectious virions in *SHFL* KO cells. Across all serial dilutions, *SHFL* KO supernatants generated more plaques of visibly greater size than WT supernatants, demonstrating that SHFL depletion strongly enhanced CVA16 propagation at the level of infectious particle output (Figure 2B).

One-step growth analysis further confirmed this enhanced replication phenotype. Infectious titers in *SHFL* KO cell lysates increased more rapidly during the early phase of infection and ultimately reached a higher plateau than those in WT cultures throughout the 48 h monitoring period (Figure 2C). Viral RNA measurements revealed a similar pattern: *VP1* RNA accumulated rapidly and reached significantly high levels in *SHFL* KO cells at the mid-to-late stages of infection (Figure 2D). Notably, infectious titers diverged between KO and WT cultures earlier than viral RNA levels, supporting that *SHFL* primarily restricts CVA16 at the post-transcriptional step after entry, rather than during viral RNA synthesis.

Immunofluorescence staining provided additional confirmation. At 24 h post-infection (hpi) at a low multiplicity of infection (MOI = 0.01), which allows visualization of differences in viral spread between genotypes, abundant CVA16 was readily detected throughout the *SHFL* KO monolayers, whereas the WT cultures contained only scattered infected cells (Figure 2E). To further establish a causal relationship between *SHFL* loss and enhanced CVA16 replication, we performed a rescue experiment by transiently re-expressing *SHFL* in *SHFL* knockout cells. *SHFL*-KO RD cells were transfected with a *SHFL* expression plasmid or an empty vector and subsequently infected with CVA16. Quantitative RT-PCR analysis revealed that re-expression of *SHFL* significantly reduced viral RNA accumulation compared with vector-transfected *SHFL*-KO cells (Figure 2F). Together, these findings suggest that *SHFL* serves as a robust intrinsic antiviral factor in RD cells, limiting both cytopathicity and viral amplification during CVA16 infection.

### 3.3. SHFL Depletion Exacerbates CVA16 Pathogenesis In Vivo

**Figure 3:**
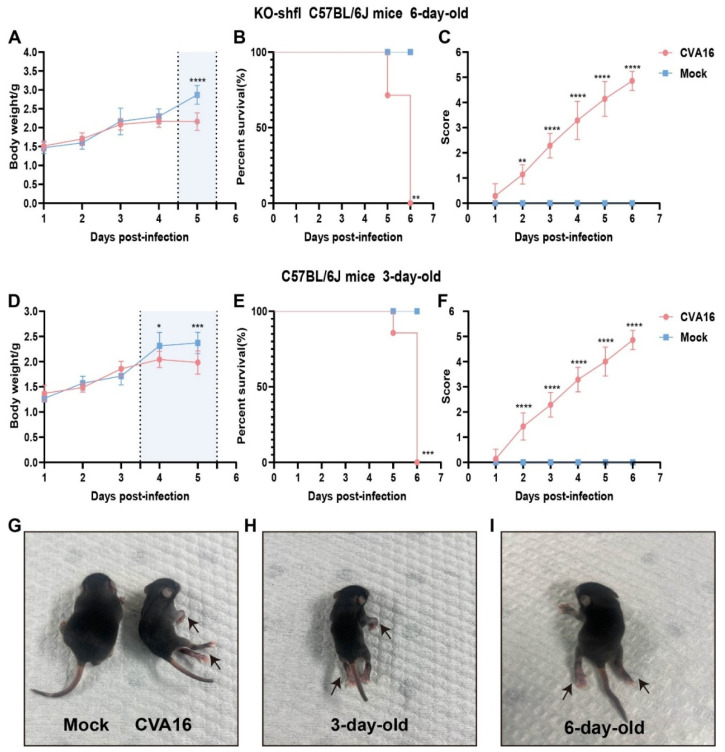
Disease progression in *SHFL* knockout and wild-type neonatal mice following CVA16 infection. (**A**) Body weight changes in six-day-old *SHFL* knockout (*SHFL* KO) and wild-type (WT) neonatal mice following CVA16 infection, expressed as a percentage of initial body weight. The shaded background indicates the time window with the most evident differences, and the vertical dashed line marks the divergence point. (**B**) Survival curves of six-day-old SHFL KO and WT neonatal mice following CVA16 infection. (**C**) Clinical scores of six-day-old SHFL KO and WT neonatal mice monitored daily after CVA16 infection. (**D**) Body weight changes in three-day-old WT neonatal mice following CVA16 infection. The shaded background indicates the time window with the most evident differences, and the vertical dashed line marks the divergence point. (**E**) Survival curves of three-day-old WT neonatal mice following CVA16 infection. (**F**) Clinical scores of three-day-old WT neonatal mice monitored daily after CVA16 infection. (**G**) Representative image of a mock-infected neonatal mouse showing normal posture and limb movement. (**H**) Representative image of a three-day-old neonatal mouse following CVA16 infection, showing mild clinical manifestations (arrows). (**I**) Representative image of a six-day-old neonatal mouse following CVA16 infection, showing severe clinical manifestations, including limb paralysis (arrows). Data are presented as mean ± standard deviation (SD; n = 6). * *p* < 0.05, ** *p* < 0.01, *** *p* < 0.001, and **** *p* < 0.0001.

To determine the in vivo relevance of *SHFL* in controlling CVA16 infection, we used a neonatal mouse model. Six-day-old *SHFL* KO mice intracerebrally inoculated with CVA16 developed rapidly progressive disease characterized by early and continuous weight loss beginning at 4 dpi (Figure 3A). Notably, neurological manifestations such as limb weakness and hunching first appeared at 3 dpi, coinciding with a clinical score of 3 or higher (Figure 3C), and uniform mortality by 6 dpi (Figure 3B). In contrast, age-matched WT littermates exhibited only mild symptoms with no mortality, indicating a strong protective role of *SHFL*. Consistent with this observation, 6-day-old WT neonatal mice infected with CVA16 under the same experimental conditions showed stable body weight, complete survival, and only minimal clinical scores compared with mock-treated controls (Figure S1).

To contextualize the susceptibility conferred by *SHFL* loss, we infected 3-day-old WT pups, which are naturally vulnerable to EV infection. Notably, their weight loss kinetics (Figure 3D), survival (Figure 3E), and neurological scores (Figure 3F) closely resembled those of 6-day-old *SHFL* KO mice, suggesting that *SHFL* deficiency functionally mimics the immunological immaturity of very young hosts.

### 3.4. SHFL Deficiency Increases Viral Loads Across Multiple Tissues

**Figure 4:**
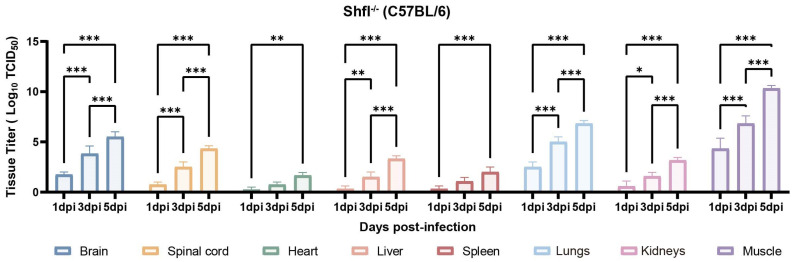
Tissue viral loads in *SHFL* knockout neonatal mice following CVA16 infection. Viral titers in indicated tissues of *SHFL* knockout (*SHFL* KO) neonatal mice at 1, 3, and 5 days post-infection (dpi). Tissues analyzed included skeletal muscle, lung, brain, spinal cord, liver, heart, spleen, and kidney. Viral loads were quantified by TCID_50_ assay using RD cells, as described in the Materials and Methods. Data are presented as mean ± standard deviation (SD; n = 5). * *p* < 0.05, ** *p* < 0.01, and *** *p* < 0.001.

Next, we quantified viral loads across the major organs of infected *SHFL* KO mice from 1 to 5 dpi. Viral titers progressively increased and were substantially higher in *SHFL* KO animals than in WT controls (Figure 4). Among the examined tissues, skeletal muscle exhibited the highest viral burden, followed by the lungs and brain, indicating preferential viral dissemination in these compartments in the absence of *SHFL*. Notably, viral titers in the heart and spleen remained relatively low and stable throughout infection.

**Figure 5:**
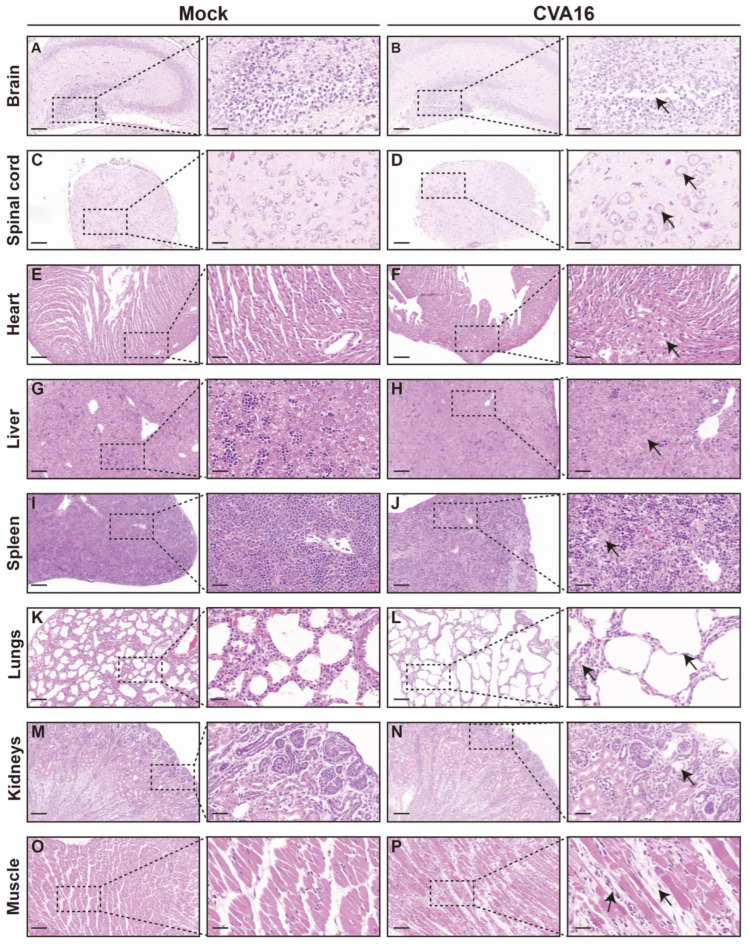
Histological analysis of tissues from mock-infected and CVA16-infected *SHFL* knockout mice. (**A**,**B**) Hematoxylin and eosin (H&E) staining of brain sections from mock-infected (**A**) and CVA16-infected (**B**) *SHFL* knockout (*SHFL* KO) mice. Low-magnification images are shown on the left, with corresponding high-magnification views of the boxed areas on the right. (**C**,**D**) H&E staining of spinal cord sections from mock-infected (**C**) and CVA16-infected (**D**) *SHFL* KO mice. (**E**,**F**) H&E staining of heart sections from mock-infected (**E**) and CVA16-infected (**F**) *SHFL* KO mice. (**G**,**H**) H&E staining of liver sections from mock-infected (**G**) and CVA16-infected (**H**) *SHFL* KO mice. (**I**,**J**) H&E staining of spleen sections from mock-infected (**I**) and CVA16-infected (**J**) *SHFL* KO mice. (**K**,**L**) H&E staining of lung sections from mock-infected (**K**) and CVA16-infected (**L**) *SHFL* KO mice. (**M**,**N**) H&E staining of kidney sections from mock-infected (**M**) and CVA16-infected (**N**) *SHFL* KO mice. (**O**,**P**) H&E staining of skeletal muscle sections from mock-infected (**O**) and CVA16-infected (**P**) *SHFL* KO mice. Arrows indicate regions with severe pathological damage. Scale bars represent 200 μm in low-magnification images and 50 μm in high-magnification images.

Consistent with viral load distributions, histopathological examination revealed pronounced organ injury in tissues with a high viral burden (Figure 5). In the CNS, infected *SHFL* KO mice exhibited hippocampal structural disorganization, neuronal necrosis, and gliosis (Figure 5A,B), with the spinal cord showing vacuolar degeneration and marked loss of anterior horn motor neurons (Figure 5C,D). The peripheral organs also showed injury. The heart exhibited early myocarditis changes (Figure 5E,F), the liver showed mild vacuolization with sporadic apoptosis (Figure 5G,H), and the spleen showed follicular atrophy with increased erythrophagocytosis (Figure 5I,J). Notably, the lungs exhibited the most severe pathology, including diffuse alveolar damage and hemorrhage (Figure 5K,L), whereas skeletal muscle developed extensive myonecrosis with inflammatory infiltration (Figure 5O,P). In contrast, kidney sections showed only minimal abnormalities (Figure 5M,N).

### 3.5. SHFL Deficiency Promotes Viral Replication and Neuropathogenesis

**Figure 6:**
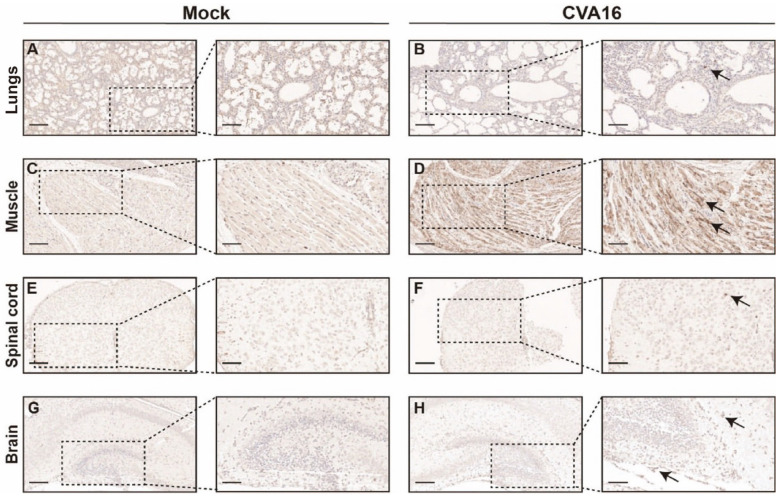
Immunohistochemical analysis of CVA16 antigen in tissues from *SHFL* knockout mice. (**A**,**B**) Immunohistochemical staining of lung sections from mock-infected (**A**) and CVA16-infected (**B**) *SHFL* knockout (*SHFL* KO) mice. (**C**,**D**) Immunohistochemical staining of skeletal muscle sections from mock-infected (**C**) and CVA16-infected (**D**) *SHFL* KO mice. (**E**,**F**) Immunohistochemical staining of spinal cord sections from mock-infected (**E**) and CVA16-infected (**F**) *SHFL* KO mice. (**G**,**H**) Immunohistochemical staining of brain sections from mock-infected (**G**) and CVA16-infected (**H**) *SHFL* KO mice. Representative low-magnification images are shown on the left, with corresponding high-magnification views of the boxed areas on the right. Arrows indicate CVA16 antigen–positive immunostaining. Scale bars represent 200 μm in low-magnification images and 50 μm in high-magnification images. Immunostaining was performed using a polyclonal anti-CVA16 antiserum; signal intensity should therefore be interpreted cautiously, particularly in tissues with low viral burden.

Immunohistochemical staining further corroborated the enhanced viral spread in *SHFL* KO mice. Abundant CVA16 antigen was detected throughout the alveolar septa and lung parenchyma (Figure 6A,B), and skeletal muscle sections showed dense intracytoplasmic viral antigens within myofibers (Figure 6C,D). Within the CNS, viral antigen was predominantly found in the spinal cord gray matter and hippocampal neurons (Figure 6E–H), indicating enhanced neurotropism in the absence of SHFL.

**Figure 7:**
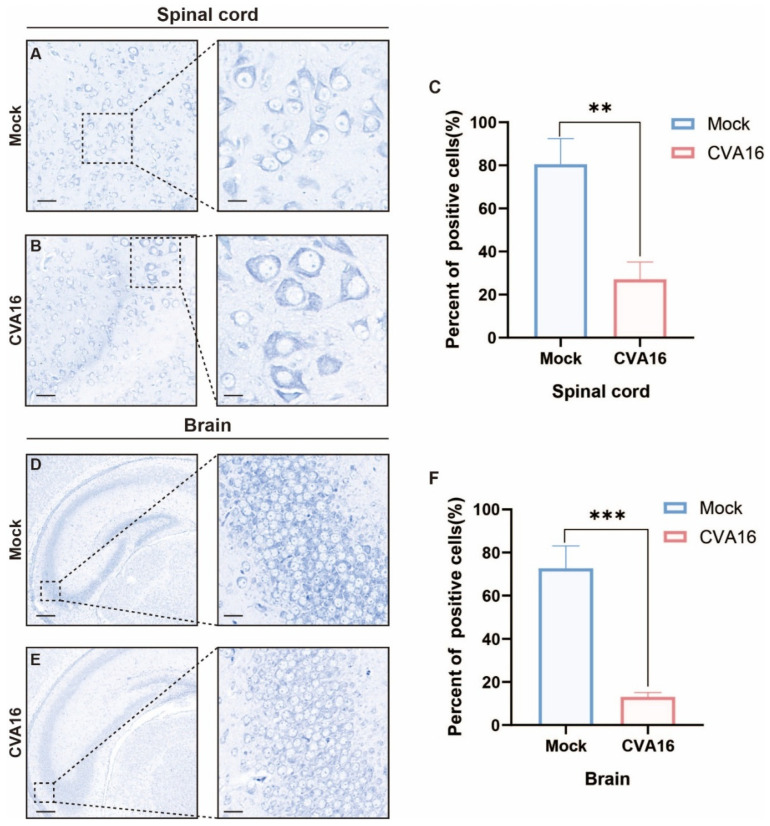
Nissl staining analysis of spinal cord and brain tissues from mock-infected and CVA16-infected *SHFL* knockout mice. (**A**,**B**) Nissl staining of spinal cord sections from mock-infected (**A**) and CVA16-infected (**B**) *SHFL* knockout (*SHFL* KO) mice. (**C**) Quantification of Nissl-positive neurons in the spinal cord. (**D**,**E**) Nissl staining of brain sections from mock-infected (**D**) and CVA16-infected (**E**) *SHFL* KO mice. (**F**) Quantification of Nissl-positive neurons in the brain. Data are presented as mean ± standard deviation (SD). ** *p* < 0.01, and *** *p* < 0.001. Scale bars represent 100 μm in low-magnification images and 20 μm in high-magnification images.

Considering the strong CNS involvement of the virus, we assessed neuropathology via Nissl staining. Infected *SHFL* KO animals showed severe neuronal injury in the spinal cord, characterized by the loss of anterior horn motor neurons, chromatolysis, and spongiform vacuolation (Figure 7A–C). Similarly, the hippocampal sections exhibited neuronal loss accompanied by pronounced gliosis (Figure 7D–F). Quantitative analysis showed that the percentage of Nissl-positive neurons in the spinal cord decreased from approximately 80% in mock-infected mice to ~25–30% after CVA16 infection (Figure 7C, *p* < 0.01). In the brain, Nissl-positive neurons were reduced from approximately 70–75% in mock controls to ~10–15% in CVA16-infected mice (Figure 7F, *p* < 0.001). These results suggest that *SHFL* plays a critical protective role in limiting CVA16 neuropathogenesis. Importantly, neuronal injury was evaluated based on quantitative counting of Nissl-positive neurons rather than qualitative staining intensity, thereby minimizing potential bias due to regional staining variability.

### 3.6. SHFL Modulates the Host Transcriptional Response to CVA16 Infection

**Figure 8:**
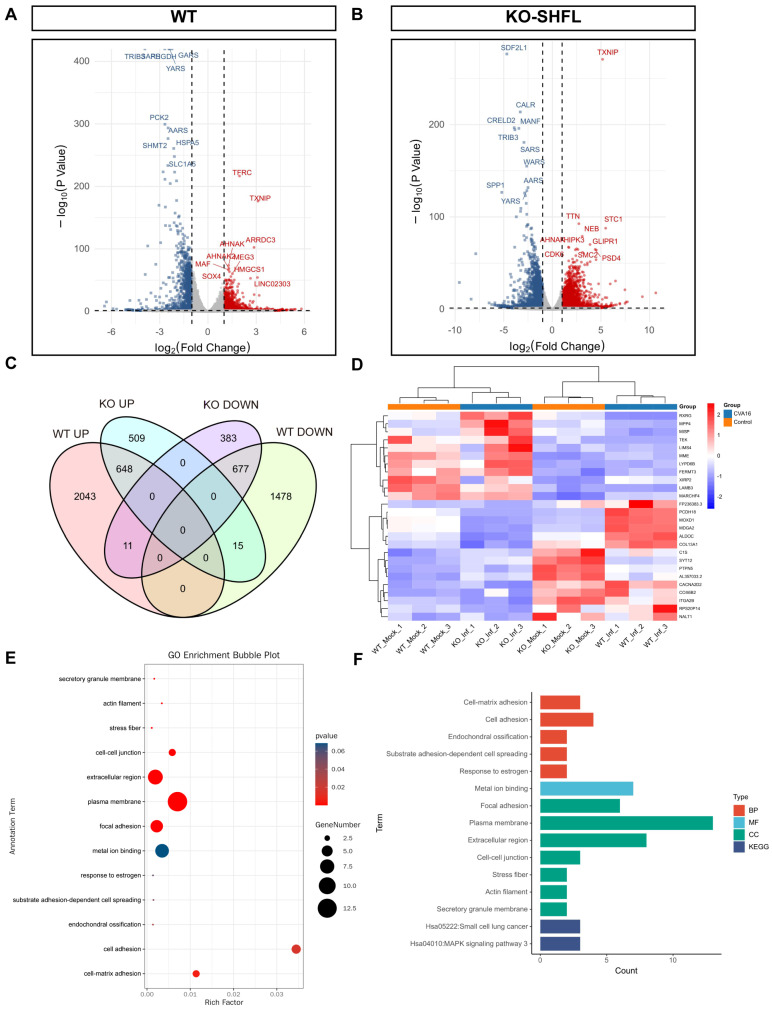
Transcriptomic analysis of WT and *SHFL* knockout cells following CVA16 infection. (**A**) Volcano plots showing differentially expressed genes in WT cells following CVA16 infection. (**B**) Volcano plots showing differentially expressed genes in SHFL knockout (SHFL KO) cells following CVA16 infection. (**C**) Venn diagram showing the overlap of upregulated and downregulated differentially expressed genes between WT and SHFL KO cells after CVA16 infection. (**D**) Heatmap showing hierarchical clustering of representative differentially expressed genes across WT and SHFL KO samples. (**E**) Gene Ontology (GO) enrichment analysis of selected differentially expressed genes. (**F**) Summary of enriched Gene Ontology (GO) and Kyoto Encyclopedia of Genes and Genomes (KEGG) pathways based on differentially expressed genes. n = 3 biological replicates per group.

Transcriptomic profiling revealed marked differences in host gene regulation between WT and *SHFL* KO cells after CVA16 infection. Under identical differential expression thresholds, WT cells exhibited 4872, whereas *SHFL* KO exhibited only 2243 differentially expressed genes (Figure 8A,B), indicating a substantially diminished global transcriptional responses in the absence of *SHFL*.

Venn diagram comparison revealed a core set of 26 genes, including membrane metalloendopeptidase, laminin subunit beta 3, FERM domain-containing kindlin 3, and *TEK*, whose expression patterns were reversed in an *SHFL*-dependent manner (Figure 8C). Heatmap clustering confirmed their distinct regulatory patterns (Figure 8D). Gene Ontology enrichment highlighted the overrepresentation of cell adhesion-related processes (Figure 8E), whereas Kyoto Encyclopedia of Genes and Genomes pathway analysis revealed enrichment in the small cell lung cancer pathway (hsa05222) and a suggestive activation tendency toward mitogen-activated protein kinase signaling (hsa04010; Figure 8F). Collectively, these results suggest that *SHFL* not only restricts viral replication but also shapes adhesion- and signaling-related transcriptional programs during CVA16 infection.

## 4. Discussion

This study identified *SHFL* as an essential host restriction factor that suppressed CVA16 replication and mitigated disease outcomes. *SHFL* expression was strongly induced following CVA16 infection and could be induced by interferon-β treatment, and genetic ablation of *SHFL* markedly increased viral replication, cytopathic injury, and tissue damage [13,14]. In neonatal mice, *SHFL* deficiency led to rapid disease progression, increased viral burden across multiple tissues, and pronounced neurological involvement [24]. Our findings suggest that *SHFL* plays central roles in limiting EV infection and protecting tissues vulnerable to CVA16-induced injury.

The enhanced susceptibility of *SHFL*-deficient mice, closely matching that of young WT animals, highlighted the contribution of *SHFL* to age-dependent antiviral defense [13]. Notably, the divergence in viral titers between *SHFL* KO and WT cells occurred as early as 2 hpi, when viral RNA levels remained comparable. This timing suggests that *SHFL* restricts viral replication at an early post-entry stage, potentially affecting viral uncoating, translation, or initiation of genome replication [18,19,20,21]. Such early restriction possibly limits subsequent viral spread in vivo. The prominent infection and pathology observed in the neurons and skeletal muscle of *SHFL* KO mice further highlight the protective relevance of *SHFL* in tissues disproportionately affected in severe EV disease. These tissue-specific effects possibly reflect differences in IFN responsiveness and ISG induction across cell types, a well-documented feature of other antiviral factors.

Our findings extend previous reports that *SHFL* functions as a broad-acting ISG with antiviral effects against diverse RNA viruses, such as flaviviruses and human immunodeficiency virus-1 [15,16,17,25]. To the best of our knowledge, this study is the first to demonstrate the fundamental role of *SHFL* in restricting EV infection both in vitro and in vivo. Previous studies suggested that *SHFL* suppresses viral translation or RNA stability, with one study reporting a post-transcriptional mechanism for EV-A71 [21]. Consistent with these observations, our data indicated a post-transcriptional action mechanism, as viral RNA accumulation diverged only later during infection, whereas infectious virus production increased much earlier in *SHFL*-deficient cells. This mechanistic distinction sets *SHFL* apart from ISGs, which primarily inhibit viral entry or RNA synthesis [12,26], and underscores its potential as a host-directed antiviral target [11]. Enhancing *SHFL* activity or expression may provide a strong barrier to viral resistance, offering an important advantage over direct-acting antivirals that target viral components and are prone to escape mutations. Recent reports suggest that *SHFL* modulates antiviral stress granule formation via phase separation mechanisms, further highlighting its therapeutic relevance [27]. Importantly, severe neurological manifestations are a shared feature among multiple enteroviruses. In addition to CVA16, echovirus 30 and EV-D68 infection models have demonstrated pronounced central nervous system and spinal cord pathology, reinforcing the relevance of *SHFL*-mediated restriction in neurotropic enterovirus infections [28,29].

This study has several limitations. First, although intramuscular inoculation reliably induced systemic and neurological diseases, it did not fully mimic natural EV transmission through the gastrointestinal tract. Second, the use of constitutive *SHFL* KO animals precluded the assessment of tissue and developmental-phase-specific effects [13], making it difficult to determine whether *SHFL* predominantly restricts CVA16 in peripheral tissues, the CNS, or both. Third, although our data suggest a post-transcriptional restriction mechanism, the precise molecular interactions, such as direct binding to viral components, modulation of host translation machinery, or regulation of RNA stability, remain unknown. Moreover, the antiviral effects of *SHFL* may vary with the immune status; however, the contribution of immune-mediated pathways could not be fully assessed using our model.

Future studies using conditional *SHFL* KO models and physiologically relevant infection routes are necessary to clarify cell type-specific antiviral functions. Additionally, mechanistic studies using ribosome profiling, RNA–protein interaction assays, and proteomic analysis are important to determine the exact steps of the CVA16 life cycle targeted by *SHFL* [18,19,20,21]. Evaluating *SHFL* activity against other EVs, such as EV-A71, CVB3, and EVD68, will also help to determine whether *SHFL* represents a broader antiviral node across the *Enterovirus* genus [22,29,30]. From a translational perspective, enhancing *SHFL* expression or promoting *SHFL*-linked antiviral pathways is a promising host-directed therapeutic strategy, particularly for infants and immunocompromised individuals who are at highest risk of severe EV disease. Future priorities include generating tissue-specific KO models, mapping spatiotemporal *SHFL* expression using single-cell approaches, and conducting high-throughput screening for small molecules that upregulate *SHFL* expression or stabilize *SHFL* function [11]. Collectively, such efforts will aid in the development of effective *SHFL*-based antiviral strategies and further enhance our understanding of host–EV interactions [31,32,33,34].

## Data Availability

The complete genome sequence of the mouse-adapted CVA16-P5 strain has been deposited at the National Microbiology Data Center (NMDC) under the accession number NMDCN00097Q6.

## Supplementary Materials

The following supporting information can be downloaded at: https://www.mdpi.com/article/10.3390/v18020192/s1. Figure S1: Body weight change, survival rate, and clinical scores of 6-day-old WT neonatal mice following CVA16 infection; Table S1: Primer sequences used in this study.

## Abbreviations

The following abbreviations are used in this manuscript:

CRISPR–Cas9	Clustered regularly interspaced palindromic repeat (CRISPR)–CRISPR-associated protein 9
CVA16	Coxsackievirus A16
IFN	Interferon
ISG	Interferon-stimulated gene
RD	Rhabdomyosarcoma
SHFL	Shiftless
