# Supplementary material for: SHFL Post-Transcriptionally Restricts Coxsackievirus A16 In Vitro and In Vivo
Source: Viruses. 2026 Jan 31;18(2):192. doi: 10.3390/v18020192 (PMC12945246; doi:10.3390/v18020192)
Supplement: Supplementary file 1 [file viruses-18-00192-s001.zip › viruses-4078791-supplementary.pdf]

Supplementary Table S1. Sequences of primers used in this study.

Gene Name	Primer Sequence (5' to 3')	Application
SHFL	F: CACCGTGCAGCCTCACCGTACACGA	RT-qPCR;
	R: CACGTCGGAGTGGCATGTGCTCAAA	CRISPR sgRNA
MX1	F:GTTTCCGAAGTGGACATCGCA	RT-qPCR
	R:CTGCACAGGTTGTTCTCAGC	
OAS1	F:TGTCCAAGGTGGTAAAGGGTG	RT-qPCR
	R:CCGGCGATTTAACTGATCCTG	
ISG15	F:CGCAGATCACCCAGAAGATCG	RT-qPCR
	R:TTCGTCGCATTTGTCCACCA	
RNaseL	F:GACACCTCTGCATAACGCAGT	RT-qPCR
	R:AGGGCTTTGACCTTACCATACA	
CVA16-VP1	F: ATGGCAGCCCAAAAGAAAC	RT-qPCR
	R: GATTGTCACCATAAGCAGCCA	
GAPDH	F: GGAGCGAGATCCCTCCAAAAT	RT-qPCR
	R: GGCTGTTGTCATACTTCTCATGG	(control)

F, forward primer; R, reverse primer.

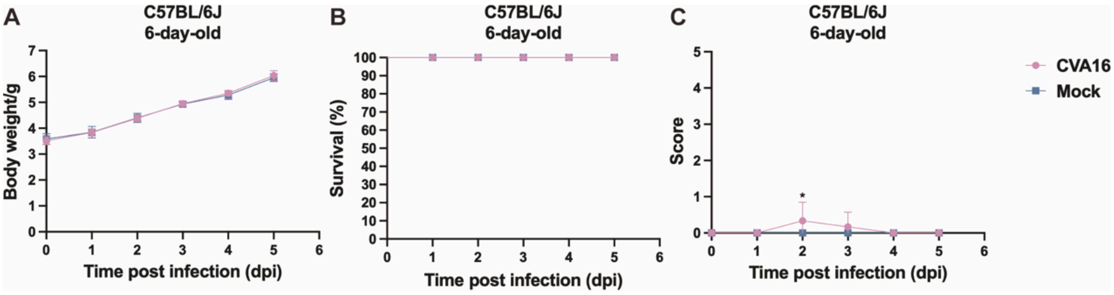

Figure S1. Disease monitoring of 6-day-old WT neonatal mice infected with CVA16. (A) Body weight changes of 6-day-old C57BL/6J wild-type (WT) neonatal mice after CVA16 infection or mock treatment. (B) Survival curves of 6-day-old WT neonatal mice after CVA16 infection or mock treatment. (C) Clinical scores of 6-day-old WT neonatal mice monitored daily following after CVA16 infection or mock treatment. The data is presented as mean ± standard deviation (SD). *$P < 0.05$.
